# Plant Methods reviewer acknowledgement 2013

**DOI:** 10.1186/1746-4811-10-4

**Published:** 2014-02-04

**Authors:** Brian G Forde, Michael R Roberts

**Affiliations:** 1 Centre for Sustainable Agriculture, Lancaster Environment Centre, Lancaster University, Lancaster LA1 4YQ, United Kingdom

## Abstract

**Contributing reviewers:**

The *Plant Methods* editorial team would like to thank all of our reviewers for their assistance with peer review in 2013.

## 

Hiroshi Abe

Japan

Comar Alexis

France

Miguel Aranda

Spain

Patricio Arce-Johnson

Chile

Michael Axtell

United States of America

Deborah Barton

Australia

Tobias Baskin

United States of America

Gerrit Beemster

Belgium

Kenneth Wayne Berendzen

Germany

Bettina Berger

Australia

Kenneth Birnbaum

United States of America

Jan Willem Borst

Netherlands

James Bradeen

United States of America

Martin Broadley

United Kingdom

Lorenz Bülow

Germany

Jeff Cain

United States Minor Outlying Islands

Maria Jesús Cañal

Spain

Eva K. Chan

United States of America

Feng Chen

United States of America

Alice Cheung

United States of America

Peta Clode

Australia

Nicola Cottee

Australia

Justyna Cybulska

Poland

Andrew Dare

New Zealand

Arthur R. Davis

Canada

Dominique Derome

Switzerland

Radhika Desikan

United Kingdom

Julien Diener

France

Lloyd Donaldson

New Zealand

Yuri Drygin

Russian Federation

Colin Eady

New Zealand

David Edwards

Australia

Matthias Erb

Germany

Abraham Escobar-Gutiérrez

France

Yu-Qi Feng

China

Emilio Fernandez

Spain

Britta Forster

Australia

Fabrice Franck

Belgium

Andrej Frolov

Germany

Dongying Gao

United States of America

Paul Gauthier

United States of America

Alan Gay

United Kingdom

Christiane Gebhardt

Germany

Erin Gilchrist

Canada

Simon Gilroy

United States of America

Sol Green

New Zealand

Peter Gregory

United Kingdom

Bo-Keun Ha

Korea, South

Neil Hall

United Kingdom

Yuepeng Han

China

Johannes Hanson

Sweden

Richard Haslam

United Kingdom

Ingo Hein

United Kingdom

Glenn Hicks

United States of America

Takumi Higaki

Japan

Thomas Higgins

Australia

Dirk Hincha

Germany

Naoki Hirotsu

Japan

Noel Holbrook

United States of America

Stefan Hortensteiner

Switzerland

Franklyn Howe

United Kingdom

Larry Hufford

United States of America

Klaus Humbeck

Germany

Andrzej Jerzmanowski

Poland

Jose Jimenez-Berni

Australia

Julia Kehr

Spain

Karin Köhl

Germany

Friedrich Kragler

Germany

Felix Krueger

United Kingdom

Patrick Krysan

United States of America

Benoît Lacombe

France

Jianfeng Li

United States of America

Xiaokun Li

China

Hsiang-chin Liu

Taiwan

Yuqiang Liu

China

Guillaume Lobet

Belgium

Chiang-Shiong Loh

Singapore

Rajjou Loïc

France

Desmond Lun

United States of America

Jingchu Luo

China

Samuel Marguerat

United Kingdom

Alan Marshall

Australia

Jaideep Mathur

Canada

Margaret McCully

Australia

Charles Melnyk

United Kingdom

A Harvey Millar

Australia

Nathan Miller

United States of America

Jayma Moore

United States of America

Katie Moore

United Kingdom

Kengo Morohashi

United States of America

Bernd Müller-Röber

Germany

Bertrand Muller

France

Andreas Nebenführ

United States of America

Massimo Nepi

Italy

Jeroen Nieuwland

United Kingdom

Anne Osbourn

United Kingdom

Linda Pardo

United States of America

John Patrick

Australia

Matthew Paul

United Kingdom

Andrea Pitzschke

Austria

Nicholas Provart

Canada

Om Rajora

Canada

R George Ratcliffe

United Kingdom

Ruben Rellan-Alvarez

United States of America

Ralf Reski

Germany

Fran Robson

United Kingdom

Daniele Rosellini

Italy

Fabrice Roux

France

Diego Rubiales

Spain

Elena Rukavtsova

Russian Federation

Silvio Salvi

Italy

G Eric Schaller

United States of America

Hans Schnyder

Germany

Alexander Schulz

Denmark

Jorg Schwender

United States of America

Peter Shaw

United Kingdom

Jesse Silverberg

United States of America

Touradj Solouki

United States of America

Stephen Streatfield

United States of America

Ram Subramanian

United States of America

Yukihiro Sugimoto

Japan

Teruaki Taji

Japan

Petr Tarkowski

Czech Republic

Guillaume Tcherkez

France

Konstantinos Theofilatos

Greece

Colin Turnbull

United Kingdom

Shoko Ueki

Japan

Elizabeth Vierling

United States of America

Tri Vuong

United States of America

Gerhard Wanner

Germany

Donald Weeks

United States of America

Fang Wei

China

Clifford Weil

United States of America

Sue Welham

United Kingdom

Ruth Welti

United States of America

Randall Weselake

Canada

James Westwood

United States of America

Weilong Xie

Canada

Yan Xiong

United States of America

Bing Yang

United States of America

John Yarbrough

United States of America

Alper Yilmaz

Turkey

Tadakatsu Yoneyama

Japan

Ji Zhou

United Kingdom

Xiaobo Zou

China

